# Focused Delivery of Chemotherapy to Augment Surgical Management of Brain Tumors

**DOI:** 10.3390/curroncol29110696

**Published:** 2022-11-17

**Authors:** Yusuf Mehkri, Samuel Woodford, Kevin Pierre, Abeer Dagra, Jairo Hernandez, Mohammad Reza Hosseini Siyanaki, Mohammed Azab, Brandon Lucke-Wold

**Affiliations:** Department of Neurosurgery, College of Medicine, University of Florida, 1505 SW Archer Rd, Gainesville, FL 32608, USA

**Keywords:** focused chemotherapy, brain tumors, drug delivery

## Abstract

Chemotherapy as an adjuvant therapy that has largely failed to significantly improve outcomes for aggressive brain tumors; some reasons include a weak blood brain barrier penetration and tumor heterogeneity. Recently, there has been interest in designing effective ways to deliver chemotherapy to the tumor. In this review, we discuss the mechanisms of focused chemotherapies that are currently under investigation. Nanoparticle delivery demonstrates both a superior permeability and retention. However, thus far, it has not demonstrated a therapeutic efficacy for brain tumors. Convection-enhanced delivery is an invasive, yet versatile method, which appears to have the greatest potential. Other vehicles, such as angiopep-2 decorated gold nanoparticles, polyamidoamine dendrimers, and lipid nanostructures have demonstrated efficacy through sustained release of focused chemotherapy and have either improved cell death or survival in humans or animal models. Finally, focused ultrasound is a safe and effective way to disrupt the blood brain barrier and augment other delivery methods. Clinical trials are currently underway to study the safety and efficacy of these methods in combination with standard of care.

## 1. Introduction

There has been no significant improvement in the survival of patients with primary brain tumors although there has been extensive development of drug delivery methods to the central nervous system (CNS). Therapies such as surgical resection, radiotherapy, and chemotherapy have improved the survival in certain tumors, such as medulloblastoma, while glioblastomas still have a poor prognosis. The different responses to pharmacotherapy may be due to differences in the blood brain barrier penetration and tumor microenvironment. To overcome these barriers, several drug delivery methods, such as nanoparticles, convection enhanced delivery, focused ultrasound, and intranasal delivery have been designed [1]. These drug delivery systems are designed to introduce therapeutic substances to the CNS, while controlling the rate, time, and region of its release.

## 2. Evolution of Drug Delivery to the Brain

**Figure 1:**
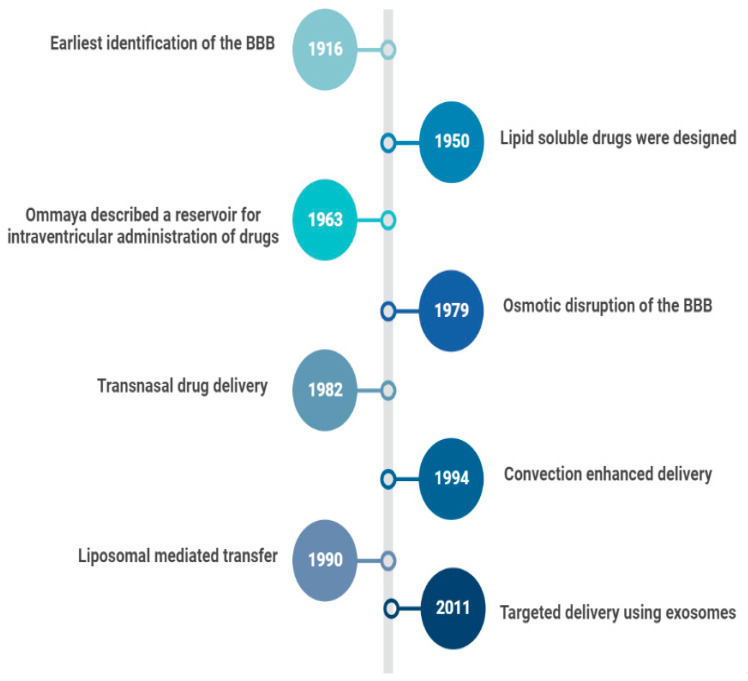
History of CNS Drug delivery evolution.

In 1914, it was reported that salvarsan and neosalvarsan both used for syphilis did not enter the brain, via blood, after IV administration [2]. By 1950, lipid soluble drugs, such as tricyclic antidepressants were designed, which traversed the blood brain barrier (BBB) well [3]. The earliest understanding and definition of the BBB was established by McIntosh and Fildes in 1916 [4]. In 1972, the lipid solubility effects on the BBB transport were further explained by Oldendorf [5]. In 1963, the first drug delivery method through the lateral ventricle was developed by Ommaya who designed a reservoir for the administration of intrathecal antibiotics [6]. In 1979, osmotic disruption of the BBB was introduced for drug delivery [7]. In 1982, transnasal drug delivery was identified as a method to bypass the blood brain barrier [8]. By 1994, different transcranial delivery methods started to emerge, such as intracerebral implants and convection enhanced delivery [9,10]. In 1986, BBB mediated transcytosis was used, and antibodies targeting BBB associated receptors to enhance the specific targeting of therapies were developed [11,12]. In 1990, a liposomal mediated transfer was first described as an advance into the history of CNS drug delivery [13]. In 2001, microbubble and ultrasound focused disruption of the BBB was first used as a drug delivery enhancing method [14]. In 2011, the targeted delivery of siRNA to the brain was established using exosome vesicles [15]. Figure 1 is an outline of the history of CNS drug delivery.

## 3. Methods of Drug Delivery

### 3.1. Non-Viral Nanoparticles

Nanoparticles have long been used as tools for drug delivery. They can penetrate the leaky tumor capillaries due to their small size, explained by their enhanced permeability and retention effect [16]. Transporter ligands and receptors can direct the uptake of the nanoparticle through the BBB to a specified target [16]. The ligand is not of a therapeutic value, but it facilitates the proper targeting and delivery of the drug [16]. A well-known example is the low-density lipoproteins undergoing transcytosis through brain endothelial cells [17]. In one phase 2 clinical study, investigating enhanced penetration in gliomas, a cell penetrating peptide was conjugated with ANG1005, angiopep-2 paclitaxel conjugate [18]. Angiopep-2 is a ligand fashioned to bind to a low-density lipoprotein receptor-related protein-1 (LRP-1), which enhances LRP-1-mediated transcytosis [18]. In experimental trials, it improved the outcome in glioblastoma mouse models [19].

### 3.2. Exosomes

Different cells secrete small extracellular vesicles called exosomes. They are stable and can remain in the circulation for a long time. Those isolated from brain endothelial cells regulate the process of transport across the BBB [20]. Exosomes have been utilized to carry small molecules and nucleic acids. In addition to carrying useful materials, exosomes have adhesive proteins on their surface [21]. To combat tumor development, an exosome containing siRNA specific vascular endothelial growth factor (VEGF) was able to release its content into brain tumors of zebrafish. VEGF expression was significantly impacted by the siRNA [22]. Additional studies suggest that brain endothelial derived exosomes may bypass the BBB and noninvasively deliver chemotherapeutic drugs [23]. 

### 3.3. Active Transport through Blood-Brain Barrier 

Amino acids can cross the BBB using different carriers. Linking drugs to these amino acids can help in the delivery of these drugs. Methotrexate (MTX)-lysine conjugate was designed to improve the permeability of the BBB to methotrexate [24]. Methotrexate was transported using the same mechanism of lysine amino acid transport. Although peptide carriers are promising, the synthesis process and optimization is exceedingly complex. More work must be performed to improve the ease of use before sustained usage can be accomplished. Additionally, prodrugs (esters) are potential BBB drug delivery methods. Furthermore, dimers can be utilized as they can carrier the drug of choice and another versatile compound. Lastly, due to their diversity and optimization, nanoparticles (organic or inorganic) possess a tremendous promise in being coupled with drug delivery [25].

### 3.4. Microbubble-Enhanced

Microbubble-enhanced delivery is a noninvasive technique, which increases the permeability of the BBB for different treatments. It was found to reduce the expression of different junctional proteins that are responsible for the integrity of the BBB without damaging brain tissue [26]. This technique was reported to increase the local brain concentration of doxorubicin and to enhance drug passage across both the BBB and the blood brain tumor barrier (BBTB) [27]. In early 2000, Hynynen and co-workers used MRI-guided US in combination with a microbubble to open the BBB [28]. Adding the microbubble technique mitigated the brain injury caused by the ultrasound waves. Moreover, Herceptin, the monoclonal antibody used to treat breast cancer brain metastasis was reported to efficiently reach the brain when US and microbubbles were used simultaneously [28].

### 3.5. Convection Enhanced Delivery (CED) 

In the early 1990s, Edward Oldfield and colleagues designed CED [29]. Notably, CED alters the permeability of the BBB and allows for subsequent targeted drug delivery. The drug delivery is localized but invasive [30]. Utilizing CED involves one or more intracranial catheters connected to an external infusion pump, enabling therapeutic substances to be delivered to the target tissues via a pressure gradient. With local infusion, the brain parenchyma receives a higher therapeutic concentration with less systemic side effects [31]. 

**Figure 2:**
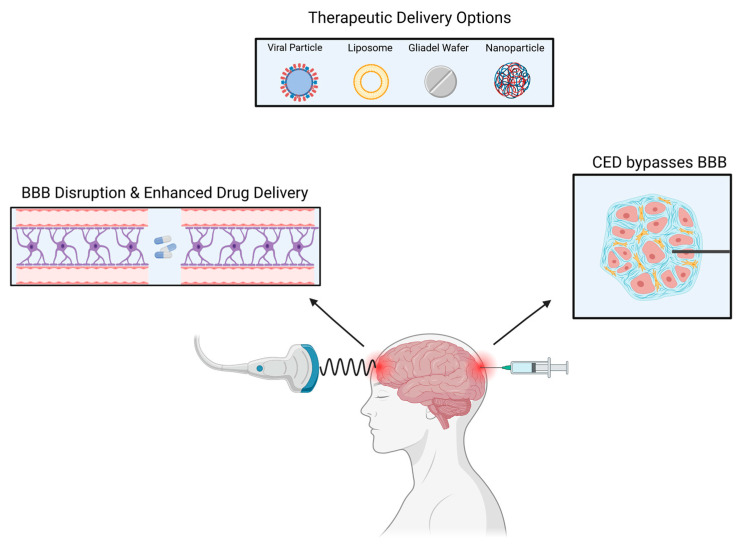
Several methods can be used to deliver drugs to intracranial tumors.

There are currently multiple ongoing clinical trials utilizing CED, most of which are for glioblastoma (GBM) and diffuse intrinsic pontine glioma (DIPG) [32]. In GBM, recurrence mainly occurs in the peritumoral area, therefore, CED utilization is vital for tumor recurrences as it perfuses different areas of the tumor [33]. CED allows for the direct delivery of the drug to the tumor bed resulting in high local concentrations with minimal systemic absorption [34] (Figure 2). 

A wide range of therapeutic elements can be delivered through CED such as chemotherapeutic agents, imaging tracers, proteins, viruses, liposomes, and nanoparticles [35,36,37]. Lastly, in addition to other limiting factors, the ideal CED drug has not yet been found with the best therapeutic index. Due to CED’s heterogeneous pressure gradient, the concentration of drugs in the treated area is not uniform, resulting in an inhomogeneous distribution. There is also an ‘intrinsic’ backflow of solutes and air bubbles adjacent to the catheter due to catheter-induced tissue damage and reflux [38]. A new avenue of research involves MRI coupled CED. The utilization of an MRI is crucial to maintain visual confirmation and to avoid complications. Additionally, to prevent reflex, a device referred to as reflux-resistant infusion cannula ensures reflux induced tissue damage is mitigated [39]. 

### 3.6. Laser Interstitial Thermal Therapy (LITT)

Laser interstitial thermal therapy (LITT) utilizes photons for the ablation of tumors to induce subsequent necrosis. LITT can disrupt the impermeability of the BBB—allowing for the enhanced delivery and efficacy of drug therapies [40]. Reportedly, LITT can increase permeability by up to 30 days post-treatment. This increased permeability allows for molecules as large as immunoglobulins to cross the BBB [41]. Notably, LITT has successfully been used in conjunction with doxorubicin to treat glioblastomas [42]. Although LITT has demonstrated a positive impact against forms of metastasis, additional research needs to specifically delineate LITTs impact on additional tumor subtypes [40,43].

### 3.7. Nanoparticles

**Figure 3:**
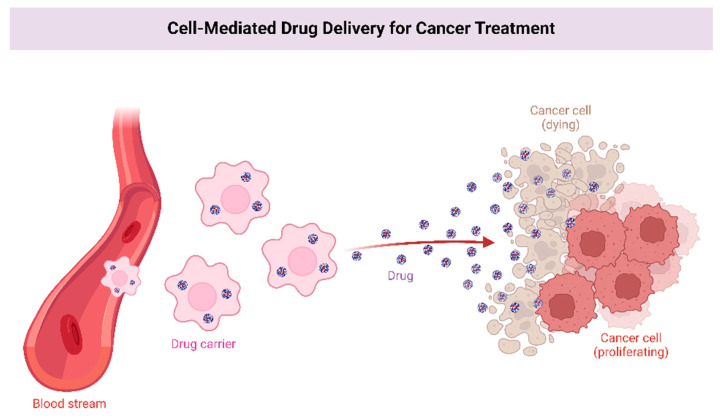
Enhanced permeability and receptor-mediated transcytosis allow nanoparticles to carry drugs with longer plasma half-lives into the brain parenchyma.

It is possible to deliver drugs efficiently using nanoparticles, which can be composed of lipids, polymers, or metallic particles. Nanoparticles can cross the BBB in various ways; through enhanced permeability and retention (EPR), endocytosis, and receptor-mediated transcytosis (Figure 3). Nanoparticles encapsulate drugs to increase the plasma half-life and to enable their entry into the brain parenchyma, as displayed in Figure 1 [44]. Several types of cancers were successfully treated with nanoparticles [45]. In EPR, the nanoparticle utilizes the leaky blood vessel of solid tumors, which can access the tumor locally [46]. Extravasation of the nanoparticle releases the encapsulated drugs into the body slowly. In most organs, nanoparticles cannot cross the normal vasculature, which reduces both peripheral and systemic toxicity [47,48]. Nanoparticles can traverse BBB leakages, making them a potential means of delivering drugs to brain tumors. Nevertheless, during clinical studies, nanoparticles could not reach tumors at therapeutic levels [49,50].

### 3.8. Intranasal Delivery

An alternative method of overcoming the BBB is intranasal delivery. The nasal cavity allows easy access without interference from the BBB to the brain parenchyma. Drugs are transported from the neuroepithelium of the nasal cavity to the central nervous system, paracellularly, transcellular, and neuronally. However, the use of intranasal medicines is not suitable for all drugs. The bioavailability of lipophilic drugs with a low molecular weight is generally more significant than hydrophilic drugs charged with a charge. For example, improving drug bioavailability with liposomes, cyclodextrins, and nanoparticles is possible. Furthermore, nose delivery of drugs avoids first-pass metabolism, thereby preserving their effectiveness [51].

Clinical trials using intranasal delivery of drugs have only yielded limited results. Malignant gliomas have been treated with perillyl alcohol intranasally. Perillyl alcohol administered four times daily resulted in 45% of cases surviving six months without progression [52]. Non-specificity of drugs can cause toxicity when delivered intranasally. Targeting tumor cells can minimize this toxicity [53]. It is also possible to reduce the toxicity in the surrounding brain tissue by using an intranasal drug delivery with microbubble mediated FUS. When these methods are combined, drug uptake in tumor regions is increased and targeted [54].

### 3.9. Intra-Arterial Delivery

**Figure 4:**
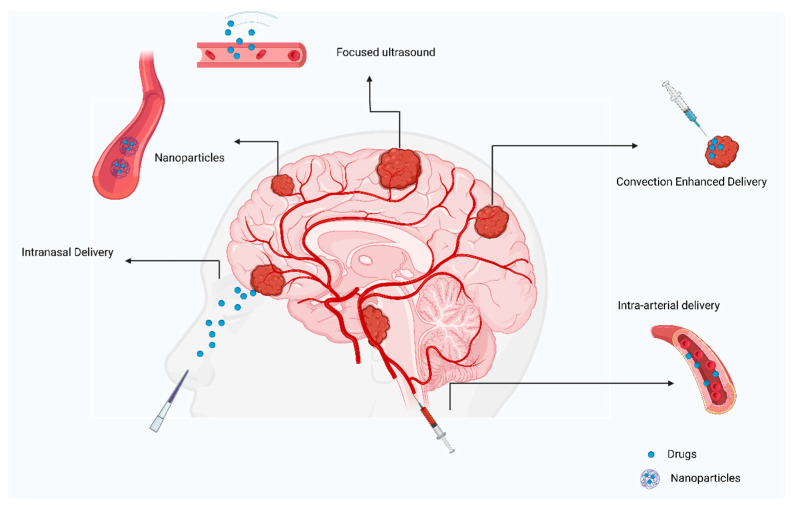
Review of current approaches to treating primary brain tumors.

Direct injection of drugs into an artery close to a tumor is referred to as intra-arterial drug delivery [55]. Drugs are released into blood vessels after cannulation in the targeted area (Figure 4). Additionally, hyperosmolar drugs, such as mannitol can be used to open the BBB on a local level [56]. However, the survival rate was not significantly improved in several clinical trials and cases. A group of patients with ependymoma were treated with cetuximab, carmustine, and bevacizumab intra-arterially, with the treatment being successful [57]. GBM patients were treated with intra-arterial drug delivery in several clinical studies. A combination of nimustine, bevacizumab, and carboplatin with other conventional chemotherapies resulted in survival rates ranging from 20 weeks to 10 months [58,59].

## 4. Pre-Clinical and Current Treatments

In this section, we review pre-clinical and current treatments used in the various focused chemotherapy modalities. The amino acid angiopep-2-paclitaxel conjugate, ANG1005, is readily taken up by the highly expressed low density lipoprotein receptor-related protein (LRP) receptors at the BBB [18,19]. ANG1005 showed a significantly enhanced brain uptake, when compared to paclitaxel alone, in rodent models with brain neoplasms [18,19,60]. There was also an increased treatment effect in humans in an ongoing clinical trial [61]. Similarly, a lysine-methotrexate conjugate showed enhanced brain delivery using the endogenous BBB lysine transport system in an in vitro and pre-clinical rodent study [22]. Rodent studies indicate that gold nanoparticles can serve as a vehicle for targeted hydrophobic drug delivery through the BBB [62,63]. This was demonstrated using doxorubicin loaded gold nanoparticles in pre-clinical studies [64,65]. Pre-clinical rodent studies have also demonstrated doxorubicin delivery using angiopep-2 decorated gold nanoparticles [66]. 

Polyamidoamine (PAMAM) dendrimers loaded with docetaxel (DTX) have shown a significantly improved glioblastoma cell death and drug delivery in the pre-clinical studies [67]. Similarly, estramustine and podophyllotoxin conjugated onto these dendrimers showed a more effective glioma cell death [68]. A G3-succinamic acid surface dendrimer conjugated with curmurin showed a tumor specific distribution in rats with implanted human glioma cells [69]. Paclitaxel linked onto G3 PAMAM dendrimers showed an increased cell brain tumor cell death and an improved porcine brain endothelial cell permeability [70]. Lastly, tamoxifen and doxorubicin linked to G4 PAMAM dendrimers have demonstrated an increased accumulation within glioma cells [71]. 

Lipid nanostructures are advantageous due to their non-toxicity when compared to other nanoparticles. A series of pre-clinical studies have demonstrated the successful use of lipid nanostructures in brain tumor models when combined with therapeutic molecules, including carmustine, doxorubicin, etoposide, siRNAs, camptothecin, edelfosine, cytarabine, rhodamine 123, pemetrexed, and miR-21 [72]. Many pre-clinical rodent and animal studies have investigated the use of liposomal drugs [73]. For example, delivery of liposomal temozolomide when combined with focused ultrasound showed improved drug delivery in rodent models of metastatic breast cancer to the brain [74]. There are several liposome drugs that exist on the market, or are currently undergoing clinical trials, which include daunoribucin for pediatric brain tumors [75] and doxorubicin for glioblastoma multiforme [76]. 

Polymetic nanoparticles have been widely used in clinical medicine. The 1,3-bis (2-chloroethyl)-1-nitrosourea (BCNU), or Gliadel^®^ wafer is an FDA approved polyanhydride implant loaded with carmustine used for the treatment of high-grade gliomas, including glioblastoma [77]. This implantable device allows for a sustained, focused chemotherapeutic release, and has improved survival rates in patients with GBM and malignant gliomas [78,79]. Poly lactic-co-glycolic acid (PLGA) is another FDA approved polymer used to encapsulate drugs. Pre-clinical rodent and cell line studies have suggested its efficacy for glioblastoma therapy when loaded with doxorubicin [80,81], bevacizumab [82], and morusin [83]. Similar studies were performed showing an efficacy against gliomas with temozolomide [84,85] and iguratimod [86] loaded with PLGAs. Chitosan is a biodegradable polymer formed by deacetylating chitin. Chitosan-coated nanoparticles containing doxorubicin successfully reduced glioblastoma growth in rodent models [87]. 

We look forward to further translational research and the outcomes of the several ongoing clinical trials [88]. There also needs to be further research regarding drug-vehicles and drug combinations and which drugs work best with specific mechanisms of delivery, such as focused ultrasound, convection-enhanced delivery, nose-to-brain delivery, and intracranial hydrogel delivery [79].

## 5. Intra-Arterial vs. Intravenous Access

Both the arterial and venous delivery of chemotherapy have been used since the beginnings of chemotherapy. Venous access preceded arterial by several years with arterial access being first attempted in 1950 [89,90]. Since then, both have had a role in the delivery of chemotherapeutics. Various trials suggested that intra-arterial delivery of chemotherapeutics would allow the administration of higher concentrations of a drug to tumors [91,92]. These trials were often fraught with complications and side effects from the intra-arterial drug administration. In 1992 Shappiro et al. conducted the first randomized trial to better understand venous vs arterial access in the treatment of malignant gliomas [82]. They found that survival was matched between the intravenous and intra-arterial groups, but that intra-arterial administration was associated with more toxicity and tissue necrosis. Under these conditions, intra-arterial administration was neither effective nor safe for the treatment of malignant brain tumors [82]. Other trials have added to our understanding of access in neuro-oncology [93,94,95]. Kochii et al. again found that intra-arterial administration of chemotherapy does not increase survival but was not associated with toxicity when compared to previous studies [93]. These results were supported by another trial with the same conclusions by Silvani et al. In addition, they found that the cost-benefit ratio of intra-arterial administration was not sufficient to consider it worthwhile in continuing. Several trials have found modest, but insignificant increases in survival [94]. In a systematic review by Cheng et al., they again found that across four trials and 460 patients, intra-arterial delivery was not superior to intravenous in terms of efficacy or overall survival [96]. At this point in time, intravenous and intra-arterial delivery of chemotherapeutics appears to be equal. Only if dosing levels and infusion techniques are improved may we see the benefits that intra-arterial delivery claims to have [97].

## 6. Ultrasound in Focused Blood Brain Barrier Disruption

The blood brain barrier is a complex and highly selective semipermeable system, mostly made up of tight connections between capillary endothelial cells. This modulates the access of peripheral molecules into the brain’s circulation [98,99,100,101]. Vascular smooth muscle cells, pericytes, immunological cells, glial cells, and brain cells are additional crucial BBB supporting cells [98,100]. Notably, lipid molecules of a particular size can passively diffuse through the BBB [100,101,102]. Due to its restrictions on drug delivery and tumor site penetration, the selective specificity of the BBB poses a significant obstacle. Numerous approaches have been investigated in the past with the aim of improving drug transport into brain tumors and transiently disrupting the BBB [102,103,104,105,106]. The use of mannitol, polymeric nano- and microparticles, radiation treatment, and convection-enhanced distribution are some methods which cause chemical disruption [102,103,104,105,106,107,108].

Despite higher drug concentrations at the target, these techniques have drawbacks, such as performing direct cerebral injections that require repeated invasive administrations [102,106,107]. Chemical BBB disruption can result in unpredictably broad BBB disruption, which can have adverse systemic implications, as well as posing a risk to normal brain parenchyma [107]. It has also been demonstrated that radiotherapy can open the BBB, although it can also be temporally unpredictable and can significantly harm healthy brain structures [108]. Outside of research protocols, these BBB opening techniques are rarely used in a neuro-oncology clinical practice. This infrequent use is due to scant clinical evidence for the effectiveness and safety of these therapies. Because of the ongoing demand for efficient BBB penetration methods for targeted tumor therapy, focused ultrasound (FUS) BBB disruption has shown promise as a potential therapeutic role

Focused BBB disruption is a noninvasive, focal, safe, and reversible procedure to treat brain tumors (oligodendroglioma, ependymoma, astrocytoma, and oligoastrocytoma (mixed glioma) [102,109,110,111,112]. Moreover, it has proven its effectiveness in neurological disorders, such as Parkinson’s and Alzheimer’s disease [113,114,115]. This technique overpowers the limitations set by the BBB and enhances the delivery of molecules into the brain that are useful for the treatment of brain disorders. To successfully deliver chemotherapy to the targeted area of the brain, FUS BBB disruption modifies the brain’s protective barrier [102,116]. This is accomplished by transmitting low-frequency ultrasonic waves through an external device to the targeted brain parenchyma [28,111,117,118,119,120]. The use of low frequency ultrasound allows for the reduction in any potential harm to the brain’s permanent tissue [28,111,112].

Low-frequency sound waves are given when lipid-coated perfluorocarbon gas microbubbles are intra-capillary infused [102,121]. In response to FUS sound waves, the BBB is altered due to consequent acoustic cavitation, rapid oscillation, high concentration of waves, and the collapse of microbubbles in the capillary walls [102]. These microbubbles further reduce the frequency of waves, which has an additive effect on BBB disruption [98,100,122,123]. As a result, the barrier becomes more permeable, making it easier for medications and therapeutic molecules to cross the BBB and enter the brain’s targeted regions [109,122]. BBB FUS is temporary and closes after 4 to 8 h, which avoids the BBB being permanently compromised, preventing neurotoxicity-related long-term negative effects [102,109,112].

**Figure 5:**
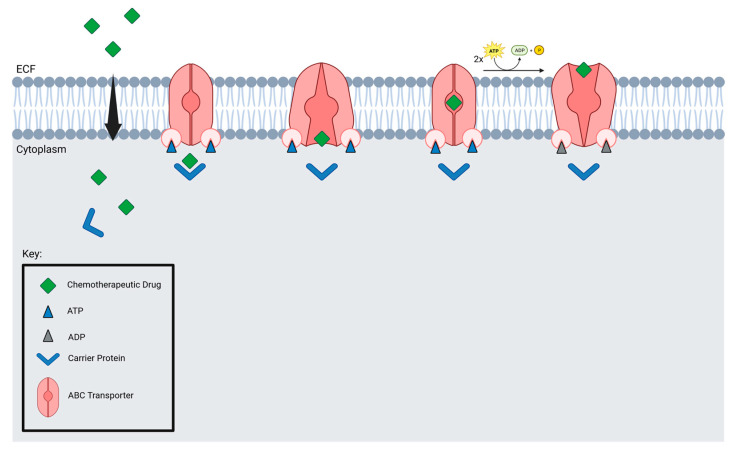
ABC transporter mechanism of action.

Microbubbles and drugs are delivered to tumors by blood vessels, which are crucial for microbubble delivery. Focused ultrasound may not be suitable for brain tumors with low vessel density. Additionally, efflux transporters prevent drugs from accumulating in the brain. Nevertheless, studies have found that FUS suppresses MDR1, resulting in an increased accumulation of drugs in tumor cells [124]. ABC transporters are found in several brain tumors, including GBM, DIPG, and medulloblastoma (Figure 5).

FUS may increase drug accumulation within these tumors. GBM and DIPG can be targeted with a noninvasive drug delivery method, such as FUS. MRI-guided FUS (MRgFUS) was performed for the first time on patients with GBM [111]. Analyzing tissue from two patients, temozolomide concentrations were 1.5 to sevenfold higher in tumors that had been sonicated than in those that had not. All patients tolerated the treatment well [111]. Several clinical studies have examined implanted ultrasound devices: CarThera (SonoCloud) was used in phase I clinical trials in conjunction with carboplatin systemic administration [125]. There are some disadvantages to these implanted ultrasound devices, including the requirement of invasive surgery, their inability to target precisely, and their suitability for superficial brain tumors. Brain cancer might be treated more effectively by combining FUS with immunotherapy. Immune cells cannot pass through the BBB because their adhesion molecules are low on CNS endothelial cells [48].

MRI-guided focused ultrasound allows for a better assessment of tissue characteristics, such as skull thickness. This allows for the precise targeting of different brain and tumor areas [102,110,113]. Prognostically, 70–80% of patients with malignant glioma die within two years of chemotherapy. Due to this dismal statistic, ample resources should be used to attempt to lengthen the survival of diagnosed patients [126]. While administering monoclonal antibodies and chemotherapeutic drugs (methotrexate and doxorubicin) in animal model pre-clinical trials, FUS BBB disruption displayed efficacy and safety [117,127,128,129,130].

According to studies using rat and mouse glioma models, FUS-induced BBB opening was linked to higher tissue TMZ concentrations, which improved tumor control rates and lengthened animal longevity [127,131]. For instance, the BBB opened with FUS, following the injection of TMZ in Fisher rats, implanted with 9-L glioma cells, was related to a higher TMZ CSF/plasma ratio, a lower 7-d tumor progression ratio, and an enhanced survival of TMZ-FUS-treated rats by 38% when compared to the controls [131]. Additionally, focused ultrasound BBB disruption enables viral gene-based therapy, tumor targeting with therapeutic nanoparticles (such as gold nanoparticles for malignant brain tumors), and the tumor’s exposure to US waves have all been further linked to an immunomodulatory role, a secondary to tumor antigen exposure and activation of heat shock proteins, which may stimulate tumor immunogenicity [102,127,132,133].

In 21 patients with recurrent GBM, a single-center trial (NCT02253212) examined the safety and effectiveness of an implantable, low-intensity pulsed ultrasound device with microbubble injection [125,134]. The BBB disruption was assessed using contrast-enhanced T1-weighted brain images, the treatment was found to be safe and without any serious side effects or carboplatin-related neurotoxicity Patients with a documented BBB disruption had a longer progression-free and overall survival time than patients without or with poor BBB disruption [134]. Another recent study by Park et al. (Clinical trial: NCT03712293) is the first study to repeatedly apply MRgFUS to the same target, while administering a chemotherapy regimen to patients with malignant brain tumors, hence demonstrating the viability and safety of repeated temporary BBB disruption using MRgFUS. This study also offers the potential for future research that assess the utility of other adjuvant chemotherapy and immunotherapy treatment deliveries, which were challenging in the past due to the intact BBB.

Studies suggest that around 10% of the pediatric population and 2.5% of adults are diagnosed with brainstem glioma (BSG) [135]. With this prevalence rate, there is a need for an absolute treatment option for brain tumors. Unfortunately, there is no ultimate solution to target and treat malignant tumors yet. While one therapy alone has not shown absolute treatment potential and survival benefits, with the advances in neurosurgical care, multiple adjuvant therapies, when used in conjunction (mostly post-surgical resection), can reduce the overall mortality rate, and lengthen the survival period after therapy [126,136,137]. Although the benefits of surgical resections of brain tumors vary with different age groups and the types of resections, the gross total resection (GTR) has a higher survival rate than a subtotal resection, with a better 5-year cancer-specific survival [109].

With technological advancement in the neurosurgical field, minimally invasive procedures, such as neuroendoscopy and endonasal endoscopic surgery are preferred to open brain surgery, because of the lesser associated complications. Moreover, adjuvant targeted irradiation, after open surgery (craniotomy), is a preferred choice to avoid the neurotoxicity, which was previously seen with whole brain radiation [137]. Overall, the treatment modalities have advanced significantly in the past several decades and now hold potential for improved outcomes with respect to longer survival rates and a better quality of life for patients with brain tumors. With availability of advanced treatment options and the enhancement of targeted drug delivery systems to the tumor vis modalities, such as FUS BBB disruption, we need clinical trials to assess the efficacy of this method in improving the outcomes of patients with brain tumors, while also assessing areas of improvement for a better clinical benefit to the patients.

A current avenue of research is the enhancement of liquid biopsies. Currently, utilizing cerebrospinal fluid (CSF) to detect tumor-derived biomarkers is non-significant. However, using MRgFUS to open the BBB could increase the concentration of tumor-derived biomarkers within the CSF. This would noninvasively allow physicians to monitor the development of a tumor [138]. Moreover, microbubble-enhanced focused ultrasound is a newly developing technology that may disrupt the BBB in a more efficient manner—allowing for the delivery of chemotherapeutic drugs [139].

## 7. Conclusions

The BBB is a complex barrier that limits options for the treatment of brain tumors. The methods most substantiated by promising data are FUS and CED. FUS allows for noninvasive, local, safe, and most importantly the reversible opening of the BBB. The utilization of FUS can also inactivate an important gene, MDR1, thus increasing the accumulation of chemotherapeutic drugs within cancer cells. Furthermore, FUS can be coupled with MRI imaging to enhance the precise drug targeting of differing brain regions. Lastly, microbubble enhancement with FUS has been used to increase the survivability of patients with GBM. CED is an invasive yet precise form of drug delivery that utilizes pressure gradients. Specifically noted for the ability to perfuse all areas of the GBM tumor, CED can isolate drug delivery without systemic absorption. Lastly, CED is noted for its versatility, allowing vectors such as chemotherapeutic agents, imaging tracers, proteins, viruses, liposomes, and nanoparticles to enter cancer cells. Other strategies that were discussed in this review, such as exosome release, active transport, and nanoparticles have limited studies supporting their efficacy and should be further investigated. To continue the research within chemotherapeutic delivery, novel processes, such as LITT and immunotherapeutic neuro-oncology, should be further explored to enhance the management of intracranial tumors in combination with surgery.
